# Ramipril-based versus diuretic-based antihypertensive primary treatment in patients with pre-diabetes (ADaPT) study

**DOI:** 10.1186/1475-2840-11-1

**Published:** 2012-01-09

**Authors:** Walter Zidek, Joachim Schrader, Stephan Lüders, Stephan Matthaei, Christoph Hasslacher, Joachim Hoyer, Claudia Zemmrich, Peter Bramlage, Claus-Dieter Sturm, W Dieter Paar

**Affiliations:** 1 Medizinische Klinik IV, Charité - Universitätsmedizin Berlin, Campus Benjamin-Franklin, Berlin, Germany; 2 St. Josephs Hospital, Krankenhausstr. 13, 49661 Cloppenburg, Germany; 3 Diabetes Zentrum, Danziger Str. 2, 49610 Quakenbrück, Germany; 4 Abteilung für Innere Medizin, St. Josefskrankenhaus, Landhausstr. 25, 69115 Heidelberg, Germany; 5 Klinik für Innere Medizin, Nephrologie, Baldingerstr., 35033 Marburg, Germany; 6 Institut für Pharmakologie und präventive Medizin, Menzelstrasse 21, 15831 Mahlow, Germany; 7 Tempestas - Institut für Medizinische Forschung, Krankenhausstr. 13, 49661 Cloppenburg, Germany; 8 Medizinische Abteilung, Sanofi-Aventis Deutschland, Potsdamer Str.8, 10785 Berlin, Germany

## Abstract

**Background:**

Previous randomized controlled trials demonstrated a protective effect of renin angiotensin system blocking agents for the development of type-2 diabetes in patients with pre-diabetes. However, there are no real-world data available to illustrate the relevance for clinical practice.

**Methods:**

Open, prospective, parallel group study comparing patients with an ACE inhibitor versus a diuretic based treatment. The principal aim was to document the first manifestation of type-2 diabetes in either group.

**Results:**

A total of 2,011 patients were enrolled (mean age 69.1 ± 10.3 years; 51.6% female). 1,507 patients were available for the per-protocol analysis (1,029 ramipril, 478 diuretic group). New-onset diabetes was less frequent in the ramipril than in the diuretic group over 4 years. Differences were statistically different at a median duration of 3 years (24.4% vs 29.5%; p < 0.05). Both treatments were equally effective in reducing BP (14.7 ± 18.0/8.5 ± 8.2 mmHg and 12.7 ± 18.1/7.0 ± 8.3 mmHg) at the 4 year follow-up (p < 0.001 vs. baseline; p = n.s. between groups). In 38.6% and 39.7% of patients BP was below 130/80 mmHg (median time-to-target 3 months). There was a significant reduction of cardiovascular morbidity and mortality in favour of ramipril (p = 0.033). No significant differences were found for a change in HbA1c as well as for fasting blood glucose levels during follow-up. The rate of adverse events was higher in diuretic treated patients (SAE 15.4 vs. 12.4%; p < 0.05; AE 26.6 vs. 25.6%; p = n.s).

**Conclusions:**

Ramipril treatment is preferable over diuretic based treatment regimens for the treatment of hypertension in pre-diabetic patients, because new-onset diabetes is delayed.

## Background

About 20% of patients with hypertension will develop type 2 diabetes in a three year period [1] and hypertension is a frequent co-morbid condition in patients with pre-diabetes and the metabolic syndrome [2,3]. While patients with pre-diabetes have an increased risk of coronary heart disease or stroke already [4], there is a further substantial increase in cardiovascular risk when diabetes actually develops [5,6] and the presence of hypertension multiplies this risk by a factor of three [7].

It was therefore important to recognize that diabetes development may be prevented by preferring blockers of the renin angiotensin system (RAS) as opposed to older treatment options such as betablockers and diuretics which promoted its development [8]. Calcium channel blockers were neutral with this respect as shown in a supplemental analysis by Lam [9]. This was learned from a number of trials reporting significant reductions in the incidence of type 2 diabetes with renin-angiotensin blocking treatment strategies in comparison to placebo, diuretics or betablockers [1,10-17]. Most of these analyses were however post-hoc and endpoints not predefined or the development of diabetes not the primary endpoint. Trials with a pre-defined new-onset diabetes endpoint were ASCOT-BPLA [14,15], VALUE [16], DREAM [1] and, more recently, NAVIGATOR [17]. While there was a significant reduction of new onset diabetes in ASCOT-BLPA (HR 0.70; 95%CI 0.63-0.78), VALUE (HR 0.77; 95%CI 0.69-0.86) and NAVIGATOR (HR 0.86; 95%CI 0.80-0.92), there was none in DREAM (HR 0.91 [95%CI 0.80-1.03]).

Because there is no large scale study on the effectiveness of blocking the RAS for the prevention of diabetes in "real world" clinical practice [18-20] we designed the ACE inhibitor-based versus diuretic-based antihypertensive primary treatment in patients with prediabetes (ADaPT) study. We aimed to verify, that the perceived benefits of using a RAS based regimen over diuretics for the treatment of hypertension in patients with pre-diabetes are also seen in clinical practice. Patients were selected based on a high risk for the development of type 2 diabetes according to the modified PreDiSc Score [21].

## Methods

### Design

ADaPT is an open, prospective, non-randomised parallel group observation at 150 office based general physicians and internists. A detailed description of the design of the ADaPT investigation has been published previously [22]. The study complies with § 67(6) of the German Drug Law and was performed under the auspices of the German Hypertension League. Ethical approval was obtained by the institutional review board of the Charité, Berlin, Germany. Patients had to provide written informed consent prior to entering the study.

### Patient population

Inclusion criteria for ADaPT were based on the PreDisc Score [21]: Age ≥ 45 years (amended, original protocol ≥ 55 years), systolic blood pressure ≥ 140 and/or diastolic blood pressure ≥ 90 mmHg, fasting glucose (IFG) level 110-125 mg/dl in venous plasma or 100-109 mg/dl in capillary whole blood and an HbA1c of 6-6.5% within the last six months. Patients with higher blood glucose levels than mentioned above, with overt diabetes or antidiabetic drug treatment were excluded as well as patients with congestive heart failure, chronic renal insufficiency, history of myocardial infarction, stroke, drug or alcohol abuse or contraindications against any of the principal drugs applied. Patients in group 1 received ramipril either as monotherapy or in combination with felodipine or another calcium channel blocker, patients in group 2 any other diuretic with or without beta-blocker-based therapy without using renin angiotensin blocking agents. Assignment of patients to either group was at the discretion of the treating physician (non-randomized). In case of inadequate response a dose increase or addition of further combination drugs was allowed within the approved labelling and in accordance with the recommendations of the European Society of Hypertension [23].

### Endpoints

The primary evaluation criterion of ADaPT was the first manifestation of type 2 diabetes (fasting glucose level ≥ 126 mg/dl/≥ 7.0 mmol/l). Blood glucose related further criteria were the deterioration of pre-diabetes indicated by an increase of HbA1c of at least 10% over baseline, an increase of fasting glucose levels and a change of HbA1c. All laboratory values were analyzed in a central core laboratory. Blood pressure related criteria were achievement of a target BP < 130/80 mm Hg and the time needed to reach target. Major cardiovascular events and mortality as well as the type and frequency of adverse or serious adverse events (AE/SAE) were also recorded.

### Statistical analyses

Data were obtained on a paper case report form and entered into a Microsoft Access 2003 database. Analyses were conducted with SPSS 17.0. Patients treated with ramipril (or ramipril-based combination therapies) and patients who received various a diuretic based treatment strategy (with the exception of ACEi or ARBs) were compared using descriptive statistics for continuous target data per treatment group and per total including the following: number of patients, means ± standard deviation, or median. The absolute and relative frequencies in percentages were determined. For a comparison of the treatment groups with respect to the incidence of specific events (e.g. patients with first manifestation of diabetes mellitus type 2 or proportion of patients with deterioration of pre-diabetes), the chi-square or the log-rank tests was used.

## Results

Between August 2004 and March 2006 a total of 2,108 patients were enrolled. Ninety-seven had to be excluded because of violations against the study protocol resulting in 2,011 patients available for the intention to treat analysis - 1,350 patients were allocated to a ramipril based and 661 patients to a diuretic based therapy. The per-protocol analysis (1,507 patients) excluded all ramipril patients, who had received any diuretic or a beta-blocking agent and all patients of the diuretic group with concomitant ACEi or ARBs. In the ramipril group 15.3% received calcium channel blocker and no patient in the diuretic group received a betablocker. Of these 1,394 patients were available for the 1 year follow-up, 1,286 for the 2 year, 1,040 patients for the 3 year and 483 for the 4 year follow-up.

### Patient characteristics

Patients enrolled (ITT, 2,011 patients) had a mean age of 69.1 ± 10.3 years, and 51.6% were female. The mean BMI was 29.9 ± 4.9 kg/m2, which meant that 41.7% were obese. With the exception of a larger proportion of female patients in the diuretic group (p < 0,001) all other characteristics, risk factors, laboratory values and co-medication were comparable in the two study groups (Table 1 &2).

**Table 1:** Patient characteristics and co-morbidity at baseline

	Intention to treat		Per protocol	
	Ramipril (n = 1,350)	Diuretic (n = 661)	Ramipril (n = 1,029)	Diuretic (n = 478)
Age (years ± SD)	69.4 ± 10.4	68.5 ± 10.3	69.4 ± 10.5	67.8 ± 10.3
Female (%)	48.7	57.5	48.5	58.4
BMI (kg/m^2 ^ ± SD)	29.9 ± 5.0	29.8 ± 4.8	29.8 ± 5.0	29.7 ± 4.9
Risk factors*				
Dyslipidemia (%)	56.4	56.6	54.7	56.9
Hyperuricemia (%)	21.7	23.0	20.5	20.5
CAD (%)	13.9	14.2	11.7	13.6
COPD (%)	6.7	4.8	6.8	5.4
Microalbuminuria (%)	6.2	5.4	6.2	5.6
Kidney disease (%)	3.0	2.9	2.8	2.1
Smoking (%)	15.6	14.4	15.6	14.4
Diabetes				
Diabetes (%)	0	0	0	0
Family history (%)	37.0	40.4	37.2	41.4
Laboratory values				
Total cholesterol (mg/dl ± SD)	222.4 ± 43.4	222.5 ± 41.8	223.4 ± 42.0	224.1 ± 41.9
HDL-cholesterol (mg/dl ± SD)	61.7 ± 16.0	61.8 ± 15.3	62.5 ± 16.1	61.7 ± 15.6
LDL-cholesterol (mg/dl ± SD)	128.6 ± 35.6	127.2 ± 34.2	129.3 ± 35.4	128.6 ± 34.1
Triglycerides (mg/dl ± SD)	169.1 ± 168.3	172.8 ± 127.5	161.8 ± 97.8	175.2 ± 137.6
Fasting blood glucose (mg/dl ± SD)	94.4 ± 21.3	95.5 ± 21.0	94.1 ± 21.3	95.7 ± 21.5
HbA1c (% ± SD)	5.6 ± 0.6	5.7 ± 0.7	5.6 ± 0.6	5.6 ± 0.7
Serum-creatinine (mg/dl ± SD)	1.0 ± 0.3	1.0 ± 0.3	1.0 ± 0.3	1.0 ± 0.3
Albuminuria (mg/g creatinine ± SD)	33.1 ± 121.6	24.6 ± 56.3	33.8 ± 131.4	19.4 ± 38.2
Urinary creatinine (mg/dl ± SD)	123.6 ± 72.7	122.7 ± 74.0	123.3 ± 72.4	120.6 ± 71.6
Cystatin-C (mg/dl ± SD)	1.0 ± 0.3	1.0 ± 0.3	1.0 ± 0.3	1.0 ± 0.3
hs-CRP (mg/dl ± SD)	5.2 ± 8.7	5.3 ± 14.2	5.1 ± 8.5	5.6 ± 16.0

Legend. * available cases only (about 12% for microalbuminuria, about 1% for the other variables); SD, standard deviation; BMI, body mass index; CAD, coronary artery disease; COPD, chronic obstructive pulmonary disease; HDL, high density lipoprotein; LDL, low density lipoprotein; CRP, C-reactive protein

**Table 2:** Antihypertensive treatment and co-medication at baseline

	Intention to treat		Per protocol	
	Ramipril (n = 1,350)	Diuretic (n = 661)	Ramipril (n = 1,029)	Diuretic (n = 478)
Antihypertensive drugs				
Ramipril (%)	76.2	0	100.0	0
CCB (%)	17.3	13.8	15.3	0
Diuretic (%)	0	72.3	0	100.0
Betablocker (%)	0.3	0	0	0
Central acting AH (%)	3.3	2.0	2.9	1.7
Alphablockers (%)	1.9	1.4	1.9	0.8
Co-Medication				
Statins (%)	19.3	17.7	17.7	16.7
Anticoagulants (%)	3.2	5.4	2.8	5.0
Thienopyridines (%)	2.0	1.4	1.7	1.5
Aspirin (%)	21.7	20.4	20.5	18.4

Legend. CCB, calcium channel blocker; AH, antihypertensives

Office blood pressure at baseline was 147.4 ± 15.9/87.3 ± 9.3 mmHg in the ramipril group and 144.6 ± 15.3/86.5 ± 9.4 mmHg in the diuretic group (p < 0.001/p = n.s.). Daytime ambulatory blood pressure was 138.2 ± 15.1/81.4 ± 10.0 with ramipril and 135.0 ± 15.0/80.4 ± 10.8 with diuretics (p = 0.001/p = n.s.; Table 3).

**Table 3:** Blood pressure at baseline

	Intention to treat		Per protocol	
	Ramipril	Diuretic	Ramipril	Diuretic
Office blood pressure				
RR systolic (mmHg ± SD)	147.4 ± 15.9	144.6 ± 15.3	147.2 ± 15.7	144.6 ± 15.3
RR diastolic (mmHg ± SD)	87.3 ± 9.3	86.5 ± 9.4	87.4 ± 9.1	86.9 ± 9.4
HR (bpm ± SD)	73.9 ± 9.4	73.0 ± 9.8	74.3 ± 9.2	73.5 ± 9.8
Daytime ABPM				
RR systolic (mmHg ± SD)	138.2 ± 15.1	135.0 ± 15.0	138.7 ± 15.3	134.7 ± 14.5
RR diastolic (mmHg ± SD)	81.4 ± 10.0	80.4 ± 10.8	81.4 ± 10.0	80.3 ± 10.4
HR (bpm ± SD)	76.3 ± 10.5	73.6 ± 11.3	76.4 ± 10.4	73.5 ± 11.5
Nighttime ABPM				
RR systolic (mmHg ± SD)	126.7 ± 17.2	122.4 ± 17.9	126.6 ± 17.3	122.4 ± 17.9
RR diastolic (mmHg ± SD)	72.4 ± 11.2	70.2 ± 11.1	72.1 ± 11.2	70.0 ± 10.7
HR (bpm ± SD)	67.3 ± 9.8	66.1 ± 9.9	67.2 ± 9.5	65.9 ± 9.9

Legend. RR, Riva Rocchi (blood pressure); HR, heart rate; SD, standard deviation; ABPM, ambulatory blood pressure monitoring

The mean fasting glucose at baseline was 94.4 ± 21.3 mg/dl in the ramipril and 95.5 ± 21.0 mg/dl in the diuretic group (p = n.s.). HbA1c was 5.6 ± 0.6% and 5.7 ± 0.7% respectively (p = n.s.). While none of the patients had diabetes at enrolment, 37.0% of ramipril treated and 40.4% of diuretic treated patients had a family history of diabetes (p = n.s.).

Patient characteristics of the per-protocol (PP) population were essentially identical to the ITT population (Tables 1, 2, 3). The only noteworthy differences were the lesser use of calcium channel blockers (0 vs. 13.8%) in the diuretic group in the PP population.

### Primary endpoint

**Figure 1:**
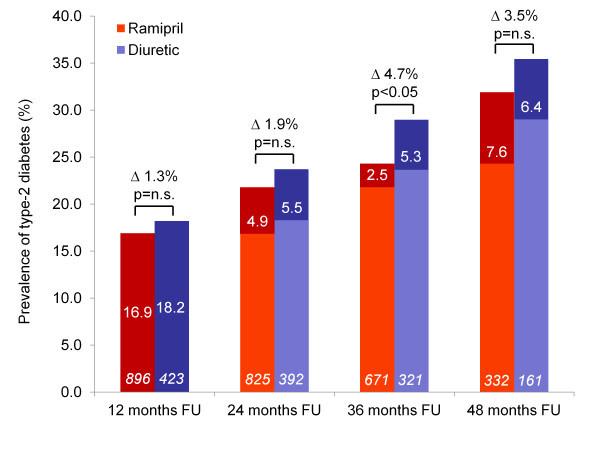
**Prevalence of new-onset diabetes in patients during the 4 year follow-up**. Legend: White numbers within columns indicate yearly incidence rates, patient numbers in italics; further differences between diabetes prevalence rates for each year are indicated together with the result of statistical testing.

The primary evaluation criterion was the first manifestation of type 2 diabetes defined as a fasting glucose level ≥ 126 mg/dl/≥ 7.0 mmol/l. The prevalence of type-2 diabetes was 0% at baseline (per exclusion criteria). Incidence rates were consistently higher in the diuretic than in the ramipril group through years 1 to 4. Strikingly however, incidence rates were 16.9 and 18.2% in the first year and between 2.5 and 7.6% in subsequent years 2 to 4. The prevalence rose continuously during follow-up (Figure 1) reaching statistical significance at a median treatment duration of three years (24.3% versus 29.0%, Δ 4.7%; p < 0.05). The difference at 4 years was largely preserved (Δ 3.5%), but became non-significant.

### Secondary blood pressure - related endpoints

**Figure 2:**
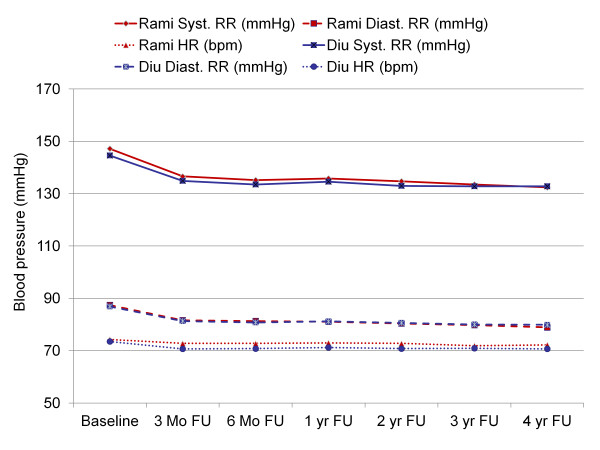
**Office blood pressure throughout the 4 year follow-up**. Legend: FU, follow-up; yr, year; RR, blood pressure (Riva Rocchi); HR, heart rate; Diu, diuretic based therapy; Rami, ramipril based therapy.

Ramipril and diuretic based antihypertensive treatments were about equally effective in lowering blood pressure (Figure 2) using office blood pressure measurement. At a mean baseline blood pressure of 147.2 ± 15.7/87.4 ± 9.1 mmHg in the ramipril and 144.6 ± 15.3/86.9 ± 9.4 mmHg in the diuretic group (Table 3) blood pressure was reduced by 14.7 ± 18.0/8.5 ± 8.2 mmHg and 12.7 ± 18.1/7.0 ± 8.3 mmHg at the 4 year follow-up (p < 0.001 vs. baseline; p = n.s. between groups).

79.8% of patients in the ramipril and 77.0% of patients in the diuretic group achieved a target blood pressure of < 140/90 mmHg at the 4 year follow-up. In 38.6% and 39.7% of patients respectively blood pressure was below 130/80 mmHg. The median time to reach a target BP of 130/80 mmHg was 3 months in either group (p = n.s.).

### Secondary glucose - related endpoints

Laboratory assessments were performed every year. No data were available on the initiation of antidiabetic medication during follow-up.

**Figure 3:**
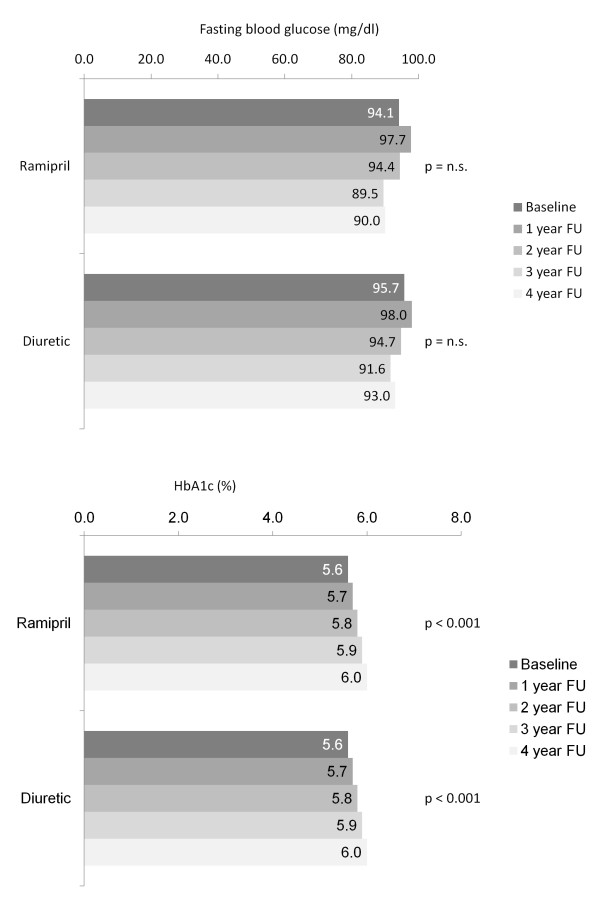
**Changes in fasting blood glucose and HbA1c trough years 1 to 4**. Legend: FU, follow-up.

Fasting glucose levels at baseline were about equal in both groups (94.1 mg/dl in the ramipril group, 95.7 mg/dl in the diuretic group; p = n.s.). Values remained rather stable over the 4 year observational period; both within group comparisons showed a trend towards slightly lower values after year 4 in the ramipril group but were not significant (Figure 3, upper panel).

HbA1c values at baseline were 5.6 ± 0.6 in the ramipril and 5.6 ± 0.7 in the diuretic group (p = n.s.) and remained virtually similar between groups throughout the 4 year observation. There was however a gradual deterioration in 0.1% steps per year, resulting in an HbA1c of 6.0 ± 0.7% in both groups at 4 years (Figure 3, lower panel). 34.9% of patients had an increase of > 10% of the HbA1c in the ramipril and 34.8% in the diuretic based group at year 4. On the contrary 7.2% of patients had a decrease of > 10% of the HbA1c in the ramipril and 6.2% in the diuretic based group at year 4. This mostly related to patients with a high HbA1c at baseline.

### Microalbuminuria

Albuminuria levels at baseline were higher in the ramipril group compared to the diuretic group (33.8 ± 131.4 vs.19.4 ± 38.2 mg/dl; p < 0.01). After 4 years follow up albuminuria was slightly reduced in the ramipril group (23.6 ± 67.0 mg/dl; p < 0.05 vs. baseline; -30%), while it was increased in the diuretic group (44.3 ± 255.4 mg/dl; p < 0.01 vs. baseline; +128%).

### Further secondary outcomes and adverse events

**Figure 4:**
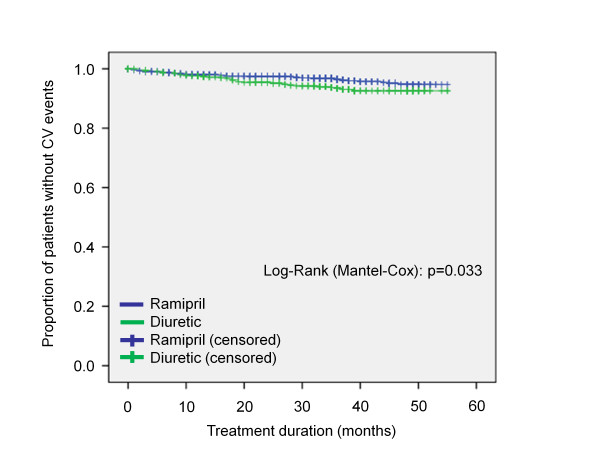
**Freedom from cardiovascular events**. Legend: CV, cardiovascular events and death.

The difference in total mortality (2.0% with ramipril vs. 2.9% with diuretics) as well as differences in major cardiovascular events (3.7% with ramipril vs. 5.0% with diuretics) including first manifestation of symptomatic coronary heart disease, peripheral arterial occlusive disease and cerebrovascular events was similar in both treatment groups (p = n.s.), while showing a trend for lower rates in the ramipril group. In a post hoc Kaplan-Meier analysis grouping both mortality and cardiovascular events, the difference between the ramipril and the diuretic treated group became significant in favour of ramipril (p = 0.033) (Figure 4).

Serious adverse events (12.4 vs. 15.3%; p = 0.05) were more frequent in the diuretic based treatment group (all adverse events 25.6 vs. 26.6%; p = n.s.). The most frequent classification as per MedDRA system organ class (Table 4) for serious adverse events were cardiac disorders (24.4 vs. 25.7%; p = n.s.) followed by neoplasms (18.5 vs. 14.9%; p = n.s.), general disorders and administration site conditions (19.6 vs. 25.7%; p = n.s.) and nervous system disorders (14.9 vs. 28.7%; p < 0.05).

**Table 4:** Number of patients with adverse event (AE) or serious adverse event (SAE) during survey

	Ramipril (n = 1,350)		Diuretic (n = 661)	
Type of adverse event	n	%	n	%
No AE	962	71.3	463	70.0
Any AE	388	28.7	198	30.0
Serious AE	168	12.4	101	15.3
Not serious	345	25.6	176	26.6

**MedDRA^® ^primary System Organ Classes (SOC) V. 11.1 - (Serious) Adverse Events, n = 2011 (%)**	**Ramipril**		**Diuretic**	
	**AE**	**SAE**	**AE**	**SAE**

Musculosceletal and connective tissue disorders	6.52	0.74	11.95	0.61
Infections and infestations	6.22	0.37	9.98	0.45
Metabolism and nutrition disorders	5.78	0.22	8.02	1.06
Respiratory, thoracic and mediastinal disorders	5.93	0.22	3.03	0.45
Nervous system disorders	3.41	1.70	6.66	3.78
Gastrointestinal disorders	3.48	1.04	4.24	1.36
Cardiac disorders	3.04	2.74	4.54	3.18
Vascular disorders	3.26	1.48	2.57	1.36
General disorders and administration site conditions	2.07	1.93	3.48	3.03
Injury, poisoning and procedural complications	2.22	1.04	3.18	1.36
Surgical and medical procedures	2.00	0.89	2.87	1.51
Neoplasms benign, malignant and unspecified (incl cysts and polyps)	1.93	2.22	2.12	2.12

## Discussion

Consistent with earlier studies [1,24,25] we found a significant amount of patients developing overt diabetes within the 4 year study period. We also confirmed a significantly higher rate of new onset diabetes with a diuretic based antihypertensive treatment approach compared to ramipril based therapy at the three year follow-up. This finding was reflected by a continuous increase in HbA1c over the 4 years study period. Although our results are somewhat limited by the loss of significance for differences at 4 years, we are essentially able to confirm for a real-world setting the results of previous randomized, controlled trials, in which a reduction of new-onset diabetes was reported for renin angiotensin blocking based treatment strategies in comparison to diuretics and/or betablockers in pre-diabetic, hypertensive or cardiovascular risk patients.

### New onset diabetes in perspective

The results of ADaPT are in principal agreement with prior randomized trials using ACE inhibitors or angiotensin receptor blockers (Table 5). These have been summarized in several meta-analyses [8,24-29] of which the one by Al-Mallah [24] is the most comprehensive and the one by Tocci [25] the most recent which also included the NAVIGATOR trial [17]. The results of both meta-analyses were virtually identical although Al-Mallah also included results of trials with active comparators (diuretics, betablocker, calcium channel blockers) while Tocci only considered placebo controlled trials. Al-Mallah reported a relative risk of 0.78 (95%CI 0.70-0.88) for the development of diabetes with ACE inhibitors and a relative risk of 0.8 (95%CI 0.75-0.86) for angiotensin receptor blockers versus non-RAS based treatments [24]. In the analysis by Tocci both ACE inhibitors (OR 0.8; 95%CI 0.7-1.0) and ARBs (OR 0.8; 95%CI 0.8-0.9) reduced new-onset diabetes as compared to placebo [25]. These results are in agreement with the finding of an OR of 0.83 seen in ADaPT.

**Table 5:** Results in perspective (adapted from [24])

	RAS blocking agent	Control	Number of patients	Follow-up	RR (95%CI)
SOLVD [36]	Enalapril	Placebo	4,228	3.4	0.26 (0.13-0.53)
AASK [30]	Ramipril	Betablocker/ Amlodipine	1,094	4.1	0.64 (0.45-0.90)
HOPE [31]	Ramipril	Placebo	9,297	5	0.67 (0.52-0.85)
ANBP2 [37,38]	Enalapril	Diuretic	6,083	4.1	0.70 (0.56-0.86)
ALLHAT [39]	Lisinopril	Chlorthalidone/Amlodipine	33,357	4.9	0.75 (0.59-0.94)
IMAGINE [40]	Quinapril	Placebo	2,553	2.95	0.79 (0.48-1.29)
ADaPT [22]	Ramipril	Betablockers/ Diuretic	2,011	3	0.83 (0.65-0.80)
PEACE [41]	Trandolapril	Placebo	8,290	4.8	0.85 (0.74-0.97)
CAPPP [42]	Captopril	Betablockers/ Diuretic	10,985	6.1	0.89 (0.78-1.03)
DREAM [1,32]	Ramipril	Placebo	5,269	3	0.93 (0.82-1.04)
STOP-2 [43]	Enalapril/ Lisinopril	Betablockers/ Diuretic	6,614	5	0.95 (0.72-1.26)

ALPINE [44]	Candesartan	HCTZ	392	1	0.13 (0.02-0.99)
CASE-J [45]	Candesartan	Amlodipine	4,703	3.2	0.65 (0.44-0.98)
LIFE [46]	Losartan	Atenolol	9,193	4.8	0.75 (0.64-0.88)
CHARM [47]	Candesartan	Placebo	7,599	3.2	0.81 (0.66-0.99)
SCOPE [48]	Candesartan	Placebo	4,937	3.7	0.81 (0.62-1.06)
VALUE [16]	Valsartan	Amlodipine	15,245	4.2	0.81 (0.74-0.89)
PRoFESS [49]	Telmisartan	Placebo	20,332	2.5	0.83 (0.65-1.04)
TRANSCEND [50]	Telmisartan	Placebo	5,926	4.6	0.86 (0.72-1.02)
NAVIGATOR [17]	Valsartan	Placeno	9,306	5	0.86 (0.80-0.92)

Legend. RR, relative risk; HCTZ, hydrochlorothiazide

There are two noteworthy findings in ADaPT that call for an appreciation. First the incidence rate for diabetes was about 3 times higher in the first year (17.3%) as compared to subsequent years (5.1% second, 3.4% third and 7.2% in the fourth year). This is in contrast with results of NAVIGATOR where incidence rates were about 9% each year and was not different across years 1 to 5 [17]. Further incidence rates were also low in the first year of DREAM and were progressively increasing through subsequent years [1]. Therefore the increased incidence rate in ADaPT during the first year is most likely artificial, possibly triggered by a closer surveillance of patients entering the observation. Another explanation might be the employed pre-selection of high risk patients by the PreDisc-Score in ADaPT. This might have distorted the natural time course of diabetes onset. Actually the overall diabetes incidence rate over 4 years was quite similar in ADaPT and NAVIGATOR/DREAM [1,17]. Second, while 3 year results were promising with respect to differences in incidence rates between ramipril and the diuretic group (24.4 vs. 29.5%, Δ 4.7%; p < 0.05), 4 year results were nominally but not statistically different (31.9 vs. 35.4%, Δ 3.5%; p = n.s.). This can be interpreted in two different directions: 1) The difference found at a median treatment duration of 3 years is "by chance" and the non-significant difference at months 12, 24 and 48 reflects the true effect of ramipril vs. diuretic based treatment on the incidence of diabetes. This is somewhat in line with the finding of a non-significant difference in fasting blood glucose levels in either group and the virtually identical trend for HbA1c which increased in parallel over time (p < 0.001). 2) On the contrary the 3 year result may also reflect the true effect of ramipril vs. diuretic based treatment. This is notion is supported by the nominally but statistically not different rate at year 4 (Δ 3.5%), which might be due the loss of 57.7% patients between year 3 and 4 which will limit the statistical power of this comparison. Further this interpretation is in line with the aforementioned meta-analyses [24,25] and 2 out of 3 trials reporting the effects of ramipril [1,30-32]. However, overall our study is small compared to other trials having reported outcomes with new-onset diabetes as an endpoint questioning that statistical power at year 4 is sufficient to actually capture differences between treatments.

The possibility for physicians in this real world trial to add a calcium channel blockers to the ramipril group and a betablocker to the diuretic group may be perceived to confound the principal comparison of this investigation and limit its interpretation. This is because betablockers have been demonstrated to adversely affect glucose control and calcium channel blockers, which were neutral with respect to the development of diabetes in a large network meta-analysis published by Elliott [8] and supplemented by Lam [9], have been reported to have a potentially beneficial effect on glucose tolerance and insulin sensitivity. Evidence was provided by a recent study in 17 non-diabetic patients with essential hypertension, where the third-generation calcium channel blocker azelnidipine proved to be more beneficial than second generation amlodipine [33]. While these results are of potential relevance for the interpretation of our findings, they may not fully apply: Only 15.3% of those receiving ramipril had a calcium channel blocker (mostly the second generation felodipine) added to their treatment. Moreover no patient in the diuretic group actually received an additional betablocker. On the other hand our findings may enforce the notion that a combination of an ACE inhibitor with a calcium channel blocker is actually beneficial with respect to the development of diabetes and diabetic complications as has been recently suggested by a trial comparing the combination of the third generation manidipine and delapril with delapril monotherapy or placebo [34]. In this trial, including exclusively hypertensive type-2 diabetic patients, combination treatment was able to ameliorate diabetic complications better than placebo (major cardiovascular events HR 0.17; 95%CI 0.04-0.78).

Based on these considerations we are convinced that the effects of ramipril over a diuretic based antihypertensive treatment can also be observed in clinical practice. As pointed out by Al-Mallah the number of patients to prevent one case of new onset diabetes (50-100) is too small to justify their use for simple prevention [24]. However, the use of these medications to treat hypertensive and CHF patients at risk for diabetes can be recommended. Because angiotensin receptor recently went off-patent there is no future need to justify their use based on cost-effectiveness considerations.

### Cardiovascular events and death

Any cardiovascular events were reduced with ramipril vs. diuretic based treatment in our study (p = 0.033). However, these results have to be considered with caution since they were determined from cases announced to the trialists as adverse or serious adverse events and serious underreporting of adverse events has been documented for observational studies [35]. Further a number of patients were lost to follow-up, introducing considerable uncertainty into these results. Further most of the other trials reporting incidence rates for diabetes failed to show a cardiovascular benefit. On the other hand the finding is in line with the most recent meta-analysis which included 11 trials and 84,363 patients which compared active treatment versus placebo [25]. Tocci et al found that active treatment using ACE inhibitors or angiotensin receptor blockers significantly reduced cardiovascular mortality (OR 0.9; 95%CI 0.8-1.0; p < 0.01) and had a favorable impact on non-CV mortality (OR 0.7; 95%CI 0.9-1.0; p = 0.2) as compared to placebo [25]. The findings are further also in line with the results of HOPE comparing ramipril to placebo where treatment with ramipril reduced the rates of death from cardiovascular causes (relative risk 0.74; p < 0.001), myocardial infarction (RR 0.80; p < 0.001), stroke (RR 0.68; p < 0.001), death from any cause (RR 0.84; p = 0.005), and complications related to diabetes (6.4 vs. 7.6%; RR 0.84; p = 0.03) [31]. Interestingly there was also a reduced event rate in cardiovascular high risk patients (OR 0.8; 95%CI 0.7-0.9) observed in the meta-analysis by Tocci et al. [25].

### Pre-selection of high risk patients

Given that 50 to 100 patients have to be treated with RAS blocking agents to prevent one case of new onset diabetes [24] might call for a pre-selection of patients. For this purpose the oral glucose tolerance test (OGTT) is useful but it is not convenient under daily practice conditions and the determination of fasting glucose or the HbA1c alone yields low sensitivity (62 and 58% respectively) [21].

Therefore patients were selected for ADaPT based on the PreDisc-Score, that closely correlates to the outcome of an OGTT [21]. The PreDisc-Score has a sensitivity, specificity and positive and/or negative predictive value in the range of 80% [21]. However, compared to the original PreDiSc score, we had to adapt the score based on practical experiences in the initiation phase of the study: first, HbA1c was to be measured in a central laboratory instead of the originally planned local determination due to wide variation in locally determined values. Second, the age criterion was reduced to 45 years or older in order to accelerate the inclusion rate.

The utility of the PreDisc-Score in the present has to be considered on the background of a number of variables. 1) Non-interventional studies such as ADaPT are prone to unknown bias because they are non-randomized and non-blinded. A possible result is a blurred estimate of the true effect which might underestimate differences between treatment groups. To cope with this the PreDisc-Score appears appropriate to select a patient population at high risk. 2) Despite the selection of high risk patients incidence rates within 4 years are not different from trials such as NAVIGATOR/DREAM [1,17], which might question this concept. Actually the adaption of the score to allow patients between 45 and 55 to enter the observation was never prospectively tested and has to be regarded as a limitation.

### Limitations

Limitations of our study are mainly the observational, not randomized character, which doesn't prevent bias through unknown impacting parameter. Yet the study goal was to confirm in a large cohort reflecting daily medical practice the findings of large existing randomized clinical trials. The only noteworthy differences in the baseline characteristics of both treatment groups seem to be a slightly higher proportion of male patients (51.4 vs. 42.6%) and higher percentage of calcium channel blocker use in the ramipril group (18.6 vs. 15.3%). We do not know if this affected the outcome in any relevant perspective, but such minor differences can never completely be ruled out in observational registries. As mentioned before a noteworthy limitation for interpretation of the presented outcome data is the fact, that the cardiovascular event and mortality analysis was only based on the AE/SAE reporting.

## Conclusions

The present study documents a high incidence rate for type-2 diabetes during the course of a four year observational period. Our key finding is in line with the large prospective randomized studies such as ASCOT-BPLA, VALUE, DREAM and NAVIGATOR and meta-analyses, which reported a significantly reduced incidence of new-onset diabetes with RAS based pharmacotherapy compared to diuretics, beta-blockers or placebo in pre-diabetic, hypertensive or cardiovascular risk patients. Patients at risk for the development of diabetes, who are identified with the PreDisc-Score should preferably be treated first line with ramipril rather than a diuretic or beta blocking agent for arterial hypertension.

## Authors' contributions

All authors made substantial contributions to conception and design, or acquisition of data, or analysis and interpretation of data. CZ and PB have drafted the manuscript. The other authors revised the manuscript for important intellectual content and all authors granted final approval of the manuscript to be published.

## Conflict of interests

WZ, JS, SL, SM, CH, JH, CZ and PB received consultancy fees, attended advisory boards and have held lectures for a number of pharmaceutical companies including Sanofi-Aventis. WDP declares to be and CZ to have been an employee of Sanofi-Aventis Germany. CDS has no conflict of interest to declare.
